# Experiences of operating room nurses in disaster preparedness of a great disaster in Iran: a qualitative study

**DOI:** 10.1186/s12873-023-00903-w

**Published:** 2023-11-23

**Authors:** Mohammad Rostami, Saeed Babajani-Vafsi, Arash Ziapour, Kourosh Abbasian, Mojgan Mohammadimehr, Armin Zareiyan

**Affiliations:** 1 Department of Surgical Technology, AJA University of Medical Sciences, Tehran, Iran; 2 Department of Surgical Technology, Faculty of Paramedical Sciences, AJA University of Medical Sciences, Tehran, Iran; 3 Cardiovascular Research Center, Health Institute, Kermanshah University of Medical Sciences, Kermanshah, Iran; 4 Management and Health Economics Department, AJA University of Medical Sciences, Tehran, Iran; 5 Department of Laboratory Sciences, Faculty of Paramedical Sciences, AJA University of Medical Sciences, Tehran, Iran; 6 Public Health Department, Health in Disaster and Emergencies Department, AJA University of Medical Sciences, Tehran, Iran

**Keywords:** Disasters, Nurses, Operating room nursing, Earthquakes, Disaster preparedness, Qualitative research

## Abstract

**Background:**

In recent years, Iran has encountered a growing frequency of earthquake disasters. Given that nurses constitute the largest group of healthcare providers, it is imperative that they possess adequate disaster preparedness skills, irrespective of the location or time. Despite the operating room nurses’ roles in disasters, their experiences and challenges in disaster preparedness have been overlooked. Consequently, this study aimed to investigate the experiences, challenges, perspectives, and factors influencing the disaster preparedness of operating room nurses during the 2017 earthquake in Kermanshah, Iran.

**Methods:**

The present qualitative research was carried out in Iran In 2022 utilizing conventional content analysis. The study involved conducting semi-structured interviews with 16 operating room nurses who had participated in disaster preparedness during the Kermanshah earthquake. The participants were selected using a purposive sampling approach that aimed to achieve maximum diversity. The interviews were continued until the point of data saturation was reached, and the verbatim transcripts were analyzed using conventional content analysis in MAXQDA software. To ensure the rigor of the research, Guba and Lincoln’s criteria were employed.

**Results:**

The study conducted data analysis to identify the main theme as “insufficient disaster preparedness due to a faded preparedness”, along with six major categories and eighteen subcategories related to earthquake disaster preparedness. The major categories included: knowledge and perception of preparedness for disasters; educational and training programs for disaster preparedness; equipment preparedness for disasters; managerial-organizational preparedness for disasters; clinical skills for responding to disasters; and resilient ability in disaster response situations.

**Conclusion:**

The findings of the study provide valuable insights into the dimensions of disaster preparedness in earthquake disasters among operating room nurses. Nursing managers can utilize these findings to develop effective strategies and provide support in areas such as improving knowledge and educational level, equipment preparedness, strengthening plans and managerial structures, enhancing skills, and explaining resilience strategies to improve the disaster preparedness of operating room nurses and medical organizations’ disaster response teams.

**Supplementary Information:**

The online version contains supplementary material available at 10.1186/s12873-023-00903-w.

## Background

The frequency and severity of disasters have increased throughout the world in recent years [1, 2]. These events have resulted in increased rates of mortality and injuries, destruction of infrastructure, adverse effects on a large number of people, and significant economic losses to societies [3, 4]. The United Nations Office for Disaster Risk Reduction (UNDRR; Geneva, Switzerland) has defined a disaster as “a severe disturbance in the functioning of a society that causes human, material, economic, and environmental losses beyond the society’s capabilities” [5]. In 2021, 432 natural disasters were recorded worldwide with resulting in 10,492 fatalities, affecting approximately 101.8 million people, and causing $252.1 billion in damages. Asia is the most disaster-prone continent globally, with 40% of disaster events and 66% of all affected populations residing in Asian countries [6]. Developing countries are more vulnerable to disasters due to various reasons, including inadequate programs and infrastructures [7]. Iran, as a developing country with diverse geographical locations, has been one of the most susceptible countries globally to the occurrence of disasters in recent years [8, 9]. Iran is one of the 10 most disaster prone countries in the world [10], with 93% and 50% of its land exposed to earthquakes and floods, respectively [11]. A strong earthquake with a magnitude of 7.3 on the Richter scale occurred at 18:18:16 UTC (21,48:16 local time) in the night of 12 November 2017, in Kermanshah province, an area near the border of Iran and Iraq in the west of Iran [12], which affecting 7 cities and 950 villages, damaging over 12,000 buildings, and resulting in 620 deaths and 9,388 injuries [13, 14].

Disaster preparedness is accepted as set of important measures to reduce the effects of disasters worldwide [15, 16]. Disaster preparedness is defined as “the measures taken to prepare for and reduce the effects of disasters” [15]. Inadequate disaster preparedness can result in a range of negative outcomes, including heightened psychological distress among healthcare providers, inappropriate response to disasters, compromised safety of healthcare providers, overcrowding of hospitals, and elevated mortality rates [17, 18]. Disasters often result in an increased demand for healthcare services [19]. Nurses, as the largest group of healthcare workers, play a crucial role in all phases of disaster preparedness and are essential members of healthcare teams in responding to disasters [20]. The preparedness of nurses for disasters is critical for the advancement of various functional levels of societies [21]. The special role of nurses in fighting against disasters should be considered to minimize the damages of unforeseen disasters [22]. Recent studies have investigated the role of nurses as frontline healthcare providers for disaster victims and their multiple roles in different phases of disaster preparedness, particularly in the disaster response phase [17, 23]. Poor levels of nurses’ disaster preparedness are associated with low performance and destructive effects [17]. Nurses have also played active roles in the relief of past earthquake disasters [24]. Traumatic injuries are common in disasters, and therefore, it is crucial to perform different emergent surgeries in such situations. Operating room nursing services, along with other nurses, play an important role in emergency response to disasters [25]. Then the position of operating room nurses in assisting victims and performing surgeries during disasters should be considered in disaster preparedness [26].

Learning lessons from the past disasters can lead to the development of evidence-based strategies for better preparedness, response and more appropriate disaster recovery of healthcare staff and medical system in future challenging and unpredictable disasters [27]. Exploring the experiences of nurses who have participated in disaster relief efforts provides a basis for effective disaster management and the development of plans and strategies for better preparedness. Despite numerous quantitative studies have been published on disaster preparedness, it should be noted that quantitative studies can’t adequately represent the effect of many factors on nurses’ ability to respond to disasters [28]. Studies conducted in China [29], Thailand [30] and Japan [31] have explored nurses’ experiences and perspectives on disaster preparedness in past disasters. Problems and challenges that nurses faced during the disasters, lack of disaster plans, insufficient training and personal challenges were highlighted in previous literature [32–34]. In a study conducted by Lee et al., the nurses involved in the Sichuan Ya’an earthquake mentioned deficiency in their physical and mental preparedness while sent on a mission to the disaster-stricken area [29]. In another study, participants expressed a strong sense of professional commitment to family and patients in disasters [35]. It is necessary to have a basic insight about disaster preparedness experiences in different nursing groups. The rationales for the significance of nurses’ preparedness for disasters encompass the necessity for nurses to safeguard public health, deliver prompt and efficient care to disaster victims, enhance community resilience, and ensure the continuity of healthcare services during disasters [17]. Given the importance of preparing nurses for disasters, it is necessary to conduct detailed research in various fields, prioritize the needs of vulnerable populations, evaluate the prevailing conditions, and develop relevant programs [36]. However, there is limited information on the experiences, views, and challenges of operating room nurses in earthquake disaster preparedness. The current study aimed to fill this gap by conducting a qualitative and in-depth investigation of the experiences of operating room nurses involved in disaster preparedness of 2017 earthquake in Kermanshah, Iran, which can help us to improve our knowledge and insight into the experiences, effective factors, and challenges faced by operating room nursing group in responding to potential future disasters.

## Methods

### Study design

The current study employed a qualitative research design using the conventional content analysis approach. Qualitative content analysis is a systematic method used to analyze textual data with the aim of gaining a better understanding of a phenomenon [37]. This study was conducted in accordance with the Consolidated Criteria for Reporting Qualitative Research (COREQ) guideline [38]. The analysis of interview data was used to elucidate the experiences and perspectives of participating operating room nurses and to identify the challenges they faced in the measures taken during the 2017 Kermanshah, Iran earthquake disaster.

### Participants and inclusion criteria

Participants in this study were 16 operating room nurses who worked in hospitals located in Kermanshah province, Iran, and had experience providing care services during the 2017 Kermanshah earthquake disaster. The inclusion criteria included participants to have a history of providing surgical care in the operating room for earthquake victims of Kermanshah hospitals during the 2017 earthquake, at least two years of work experience in the operating room, and willingness to participate in the study. The exclusion criteria included refusal to consent to audio recording and withdrawal from the interview.

The participants were selected using the purposeful sampling method with maximum variety to gain a more comprehensive understanding of the subjects. To achieve maximum variety, participants were selected with diversity in demographic characteristics and from several hospitals located in Kermanshah province, Iran. The final sample size was determined by the data saturation point, which was reached when no new information was obtained from the participants by continuing new interviews. To maintain data confidentiality, participants were coded as N1 to N16.

### Data collection

Potential participants who met the inclusion criteria were identified using the purposeful sampling method and invited to participate in the study. After obtaining initial agreement and explaining the study objectives, verbal and written informed consent was obtained from participants, and interview appointments were scheduled. To collect data, semi-structured face-to-face interviews were conducted between July and November 2022, with a final sample size of 16 participants determined by data saturation. The first author, M.R., who had seven years of work experience in the operating room and had completed training courses in qualitative research methods, conducted all interviews individually under the supervision of research team experts in qualitative research methods.

The interviews were conducted confidentially in a private and quiet space, typically in the rest room of the operating room staff, at a suitable appointment time for the participants. Each interview lasted between 35 and 85 min, and written field notes were taken by the interviewer during the interviews to ensure a correct description and interpretation of the data. Although data saturation was reached at the 14th interview, two additional interviews were conducted for more certainty, resulting in a final sample size of 16 participants. The interview guide used in this study was developed specifically for this study by the research team. The interview guide questions were designed based on the study objectives, according to the literature review literature review, and with considering qualitative descriptive methods [39]. Also before the main interviews, two pilot interviews were conducted to ensure the adequacy of the interview questions for achieving the study’s objectives, and minor revisions were made to the interview guide questions based on the pilot interviews. Demographic questions, such as gender, age, educational level, marital status, and years of work experience, were asked at the beginning of the interview. The interview schedule started with a general question, “Please describe your experience of participating in the disaster preparedness activities in the Kermanshah earthquake?“ and continued with questions such as:

- Please describe your experiences during the relief operation of the Kermanshah earthquake.
- What did you learn from your experiences during the disaster preparedness efforts?
- What challenges did you experience before, during, and after your work as an operating room nurse in the disaster preparedness activities in the Kermanshah earthquake?
- How did you deal with the problems you encountered?
- Based on your experiences, what components and features should be considered in the earthquake disaster preparedness of operating room nurses?
- Please tell me about the expectations and obstacles of the current status of earthquake disaster preparedness.

Probing questions were also asked during the interviews to obtain clearer and deeper details and information, such as “Can you please give an example of this?“, “Could you please explain with more details?“, “What do you mean?“, and “Is there anything else you would like to talk about it?“. The interview guide used in this study can be found in the Supplementary material section as the Interview Guide file.

### Data analysis

All interviews were conducted with the participants’ permission and audio-recorded. The researcher transcribed the interview recordings verbatim and stored them on a computer system within 24 h. The data were managed using MAXQDA-2018 software for data analysis. Data analysis was conducted simultaneously with data collection, and members of the research team were invited to participate in data analysis, coding, and theme extraction. Each interview was reviewed and analyzed by two authors, and the information retrieved was used to guide subsequent interviews with new participants. After the first interview, the process of data coding and analysis began and continued until data saturation was achieved. Data saturation is reached when no new codes or information are obtained from continued interviews, and the previously obtained codes are repeated [40–42]. Data saturation was achieved after the interview with 14 participants, and two additional interviews were conducted for more certainty. The researchers utilized the five stages proposed by Graneheim and Lundman to conduct the conventional content analysis [43]. The content analysis process involved several steps. First, the interviews were transcribed and reviewed multiple times to achieve a detailed understanding of the entire written material. Next, meaning units and primary codes were extracted from the data. The meaning units were then summarized and categorized, and an appropriate label was chosen for each category. The research team modified the subcategories and primary categories as needed. In cases of inconsistency in coding, the authors discussed all items to reach an agreement. Finally, an appropriate topic was selected to cover the categories. The nature and scope of the collected data were reviewed to ensure that each theme accurately represented the accompanying narratives, and the themes were named accordingly. This process allowed for a systematic and rigorous approach to analyzing the qualitative data.

### Rigors

To ensure the trustworthiness of the study, the researchers utilized four criteria during the study process: credibility, dependability, conformability, and transferability [44, 45]. To ensure credibility, the researchers devoted sufficient time to data collection and analysis and ensured maximum participant variety, comparability of findings, long-term and continuous interaction with data, and an in-depth understanding of the experiences. Member checks were conducted with the participants to ensure the accuracy of the extracted codes and categories. To enhance the credibility of the data, the researchers utilized the member check technique, in which participants reviewed the interview contents, codes, and categories extracted from the interviews to ensure an accurate representation of their actual experiences. Additionally, the peer review technique was used to include the opinions of qualitative experts on the research team in conducting the study, conducting interviews, categorizing and analyzing the data. To ensure dependability, the researcher collected reflexive notes during the study process, and all study steps were noted and recorded. The research team collaborated as auditors to check the process, raw data, codes, and subcategories. To check conformability, the researchers shared reflective notes on the research topic, which allowed them to acknowledge their prior experiences and understanding of the phenomena. Furthermore, throughout the study, the researchers conducted conscientious reflective thinking to examine individual opinions and thinking methods. These measures ensured the trustworthiness of the study and enhanced the credibility and applicability of the findings. Member checks were conducted by returning the interview text and summary of the results to six participants to confirm the accuracy of the findings. Additionally, two qualitative researchers in the research team checked the validity of the data collection and analysis process. To ensure the transferability of the findings, efforts were made to provide clear and distinct explanations of the context, sampling with maximum variety of participants, representative data collection and analysis process, as well as a rich and strong reporting of findings along with appropriate quotations.

### Ethical considerations

The present study followed the Helsinki Declaration and was approved by the Ethics Committee of AJA University of Medical Sciences (ethics code: IR.AJAUMS.REC.1401.064). Prior to the interviews, the time and place were determined by the participants. At the beginning of each interview, the researcher introduced themselves and explained the objectives and process of the study to the participants. The participants were assured of the confidentiality of the information they provided, and it was emphasized that there would be no negative personal or professional consequences for their participation. The personal information and identity of the participants were kept confidential. Oral and written informed consent was obtained, including a description of the purpose of the study and a guarantee of confidentiality. The participants were informed that their cooperation was voluntary and that they had the right to withdraw from the study at any stage. Interview data were retained confidentially, without the details of the participants, by the interviewer, with a printed version in a locked file cabinet and an electronic version in a password-protected folder. Research records will be retained for 5 years after the completion of the research.

## Results

### Characteristics of participants

The study included 16 operating room nurses as participants. The age of the participants ranged from 28 to 50 years, with an average age of 39.93 ± 7.31 years. The average work experience of the participants was 17.78 ± 8.28 years. Thirteen participants (81.25%) had a Bachelor’s degree, one had an associate degree, and two had a master’s degree. The majority of the participants were married, with five (31.25%) being female and the rest being male. Seven participants had managerial experience. Table 1 provides a detailed description of the demographic characteristics of the participants.

**Table 1:** Descriptive characteristic of the participants

Variable	Category	Frequency	Percentage
**Age(years)**	25–35	6	37.5
	35–45	5	31.25
	Over 45	5	31.25
**Gender**	Male	11	68.75
	Female	5	31.25
**Marital status**	Single	2	12.5
	Married	14	87.5
**Education**	Associate degree	1	6.25
	Bachelor’s degree	13	81.25
	Master’s degree	2	12.5
**Work experience(years)**	< 10	4	25
	10–20	6	37.5
	20<	6	37.5
**History of managerial position**	Yes	7	43.75
	No	9	56.25

Hospitals and operating room nurses must consider disaster situations both before and after disasters occur. After analyzing the data, one main theme, six categories, and 18 subcategories were extracted regarding the experiences of operating room nurses in earthquake disaster preparedness (see Table 2). Examples of participants’ quotes, along with codes, subcategories, categories, and the extracted main theme, are provided below.

**Table 2:** Theme, categories, subcategories, and codes obtained from interviews with participating operating room nurses

Theme	Categories	Subcategories	Codes
Insufficient disaster preparedness due to a faded preparedness	Knowledge and perception of preparedness for disasters	The operating room nursing knowledge	Deficiencies in knowledge to perform specialized tasks, The importance of updated knowledge about operating room nursing, The significance of knowledge level in performance
		Disasters specific knowledge	Familiarity with the different levels of disaster status, Understanding the types of disasters and their various dimensions, Reading disaster papers and guidelines
		Knowing and perception of duties, roles and disaster preparedness plans	Familiarity with the leaders of the incident command system and their successors, Understanding the staff duties in earthquake disasters, Being familiar with the implementation of disaster plans
	Educational and training programs for disasters preparedness	Disaster specific education	Educational courses for operating room nurses, Providing specialized training for operating room nurses, Attractive education with new teaching methods, Participating in disaster management courses
		Training programs and Exercises	Conducting training maneuvers based on operational plans, Exercises the communications and roles, Performance maneuvers and a higher level of preparedness, Conducting inter-organizational exercises
		Challenges of educational courses	Symbolic maneuvers, Non-practical and inefficient educations, Providing quick and low-content educational programs, Non-utility trainings, Importance of repetition and continuity in training programs
	Equipment preparedness for disasters	Prediction of equipment resources	Constant evaluation of equipment and deficiencies in the prevention phase, Depotting of equipment in the predicted place, Forecasting and constant preparation of equipment
		Challenges of equipment and surgical instruments in disasters	The challenge of accessing equipment, The lack of disposable equipment and surgical sets, Maintaining the sterilization of surgical instruments, Poor access to suitable equipment, The limited number of sterile surgical instruments, The long distance from the equipment storeroom, Difficulty accessing personal protective equipment, learning to work with different equipment and surgical instruments
	Managerial-organizational preparedness for disasters	Access to disaster operational plans and guidelines	Deficiencies in clear planning, non-operational plans, inappropriate implementation of plans in special irregular situations, use of specific protocols for preparation, familiarity with the principles of planning in disasters, human resource planning challenges, and organizational support for disaster management and preparedness
		Incident command system and in scene leadership	Non-arbitrary actions of staff, adherence to the command hierarchy in disasters, observation of the unity of command in performance, importance of the incident command system chart, leadership and organizational ability, having a suitable level of disaster management, management and appropriate decision-making in the scene, and lack of proper resource management in disaster situations
		Coordination in disaster management	Interpersonal and interorganizational cooperation, non-repetitive and parallel work, coordination and constant interorganizational communication, and multiple sources in management and decision-making
		Effective communication and teamwork	Awareness of communication methods between hospitals during disasters, disconnection of telecommunications during disasters, proper communication and cooperation between healthcare providers, formation of multidisciplinary teams for disaster response, team performance and familiarity with the abilities of team members, and the ability to work effectively in teams
	Clinical skills for responding to disasters	Triage skills	The importance of triaging victims of disasters, preparation to perform triage operations, performing triage as the first step of providing aid, and the challenge of performing triage in the presence of other people
		Special skills as an operating room nurse	Preparing the place for performing surgical procedures, performing professional tasks, compliance with infection control principles, quickly preparing for emergency surgical procedures, hemostasis of bleeding, suturing injuries at an appropriate speed, performing medical care for different groups, and the importance of appropriate experience in different surgical fields
		Basic nursing care skills	Performing basic nursing procedures, the ability to provide first aid, and transport and movement of victims of disasters
	Resilient ability in disaster response situations	Ethical conflicts in care provision	Limited number and sterilization of surgical instruments, performing medical care in the presence of other people, and failure to observe sterilization principles in disaster situations
		Adaptation to difficult environmental-occupational conditions in disasters	Deficiency in access to personal life facilities, the challenge of providing services for healthcare staff, high work pressure, job burnout, creativity, and providing non-therapeutic assistance
		Maintaining mental well-being in disaster situations	Stress experienced by the individual, concerns for their family, maintaining mental strength for better rescue, and controlling stress while performing duties

### Theme: insufficient disaster preparedness due to a faded preparedness

Following their experiences in earthquake disaster preparedness, the participants provided insights into various aspects of disaster preparedness. After conducting data analysis, a theme emerged which was “Insufficient disaster preparedness due to a faded preparedness”. This theme was further divided into six categories, which included Knowledge and perception of preparedness for disasters, Educational and training programs for disaster preparedness, Equipment preparedness for disasters, Managerial-organizational preparedness for disasters, Clinical skills for responding to disasters, and Resilient ability in disaster response situations.

#### Knowledge and perception of preparedness for disasters

The participants in earthquake disaster preparedness highlighted their experiences related to various aspects of disaster preparedness, including the operating room nursing knowledge level, disaster specialized knowledge, and knowing and perception of duties, roles, and disaster preparedness plans.

##### The operating room nursing knowledge

Having an adequate knowledge of operating room nursing, including the ability to perform expected professional tasks, and possessing an appropriate scientific level can lead to increased self-confidence in working under all conditions to save patients’ lives. This knowledge can also aid in decision-making for better performance in critical conditions and is related to the operating room nurses’ preparedness for disasters. However, as revealed in the interviews, limited knowledge among operating room nurses led to inadequate preparedness for the earthquake disaster. For instance, one participant stated that:

*“Acquiring knowledge through studying and learning is crucial in disaster preparedness. Having a theoretical understanding of a subject and visualizing it in one’s mind can lead to better practical performance. Individuals with specialized knowledge in their respective fields tend to have better performance in disaster situations, as evidenced by the earthquake disaster”. (Participant N.5, woman with 10 years of work experience)*

##### Disaster specific knowledge

According to the participants, having an appropriate level of knowledge and theoretical understanding of the specific concepts and requirements of disasters is crucial for disaster preparedness. Based on their experiences, the participants emphasized the importance of specialized practical knowledge in disasters, including familiarity with different phases of disasters and different levels of disaster notifications, specific knowledge on different types of disasters, knowledge of first aid and triage in disasters, familiarity with warning systems, and reading relevant articles and disaster guidelines. The participants believed that having knowledge of the different dimensions of disasters can improve performance and preparedness for an earthquake disaster, which was a challenge faced by some participants. In this regard, one participant stated:

*“Acquiring up-to-date scientific knowledge by reading relevant articles on disaster preparedness can not only help to familiarize nurses with recurring disasters but also facilitate the predictability of other disasters. This knowledge can broaden the staff’s horizons and increase their familiarity with different types of disasters, as well as their level of disaster knowledge. During the earthquake, deficiencies in disaster knowledge were faced by some nurses”. (Participant N.2, woman with 6 years of work experience)*

##### Knowing and perception of duties, roles and disaster preparedness plans

According to the operating room nurses, understanding their responsibilities and duties in preparing for earthquake disasters is crucial. This knowledge removes confusion about the performance of their duties in disasters and enables them to act with more knowledge in activities related to disaster preparedness. However, inadequate knowledge of responsibilities and scope of duties as operating room nurses, as well as for other staff involved in disaster preparedness and response, and knowledge of disaster preparedness programs were considered challenging issues by the participants. The participants stated that:

*“Prior to a disaster, it is important to focus on strengthening the knowledge aspect of personnel through the use of training booklets and pamphlets, providing charts, and explaining the duties of each personnel in a disaster. This approach can ensure that personnel are aware of their specific duties and the duties of managers and officials in the event of a disaster, such as an earthquake. By doing so, confusion and disorder, which were witnessed during the earthquake, can be avoided in the future”. (Participant N.1, man with 11 years of work experience)*

#### Educational programs and training for disaster preparedness

Disaster training and educational programs were identified as another category in the earthquake disaster preparedness experiences shared by operating room nurses. Specific disaster education, training programs, maneuvers, and educational preparation challenges were among the subcategories discussed in the interviews as being important for training operating room nurses for earthquake disaster preparedness. The subcategories are described below.

##### Disaster specific education

Some participants emphasized the importance of specialized education for disasters, which can be effective in addressing the challenges faced in earthquake disaster preparedness. Providing specific education related to occupational specialization can empower operating room nurses to provide care and take specific actions related to their profession in disaster situations, which was a gap encountered by the participants in their recent experience. To ensure optimal capabilities in the stages of disaster preparedness, healthcare organizations should improve the theoretical and functional level of operating room nurses through regular education. The importance of specialized education and various methods for providing it, such as seminars, periodical conferences, video technologies, and beginning-of-shift rounds, were highlighted and discussed during the participant interviews. They described:

*“What is important and useful in a disaster is prior educations, Prior and specialized education is crucial and beneficial in disaster situations. By providing training to personnel, their performance level can be improved during disasters. Education on the use of personal protective equipment, moving the devices, management of deficiencies, infection control, specific surgical procedures, and other relevant topics can be particularly useful in disaster situations. This education can help personnel to effectively and efficiently respond to disasters and provide appropriate care to patients”. (Participant N.3, woman with 28 years of work experience)*

*“Our problem in the earthquake was that we did not pass the education; As earthquakes may not occur for several years, it is important to provide ongoing education to operating room nurses. This can be achieved through video conferences, which can demonstrate the weaknesses and strengths of disaster preparedness to nurses in the operating room. Continuous training clips should be shown several times throughout the year, rather than just for one day, to improve the experience of nurses through education”. (Participant N.13, man with 10 years of work experience)*

##### Training programs and exercises

In most interviews, training programs and maneuvers were identified as effective and important factors in disaster preparedness for all healthcare staff, particularly operating room nurses. The interviews discussed the importance of conducting numerous periodic exercises, simulations of various disaster conditions, and operational maneuvers in the form of team and inter-organizational programs. Through these exercises and maneuvers, operating room nurses can simulate disaster relief conditions in cooperation with other medical and non-medical staff, experience and practice their roles and duties, and prepare for real disasters that may occur at unpredictable times and places. The participants stated that going through such training programs can have a positive impact on their level of preparedness for earthquake disasters. One participant said:

*“In disaster preparedness, it is important for not only operating room nurses and other medical staff but also all officials in the disaster chart to participate in training classes and maneuvers. Through exercises and maneuvers, various communications can be expanded, roles can be defined, and deficiencies for a major disaster can be identified. This can ensure that if a disaster occurs, the staff’s performance will be in the best possible condition”. (Participant N.12, man with 15 years of work experience)*

##### Challenges of educational courses

In their experience of earthquake disaster preparedness, almost all participants mentioned challenges related to educational preparedness. These challenges included the absence of training courses on disaster preparedness during university studies, the lack of specific workplace training, deficiencies in the continuity of in-service education, the lack of up-to-date and practical content in education, the symbolic nature of maneuvers and exercises, and the lack of interorganizational maneuvers. Operating room nurses found these educational challenges to be effective in their level of disaster preparedness. For instance, participants said:

*“Prior to the earthquake disaster, a series of training exercises were performed. However, the experience of being in a disaster is vastly different from training exercises”. (Participant N.11, man with 30 years of work experience)*

*“Continuous and repeated education is necessary to ensure that the contents remain in the minds of personnel and are not forgotten. Educators should receive feedback on the impact of the training, such as how much it has influenced the participants. If the education is not effective, the method of expression or the approach may need to be adjusted. Challenges have been experienced in practice where the education was not suitable, highlighting the importance of effective and appropriate education in disaster preparedness”. (Participant N.8, man with 17 years of work experience)*

#### Equipment preparedness for disasters

The preparation of equipment resources for disasters involves a set of preparations, including predicting the availability of equipment resources, working with instruments, tools, and equipment, and preparing for equipment challenges in disasters. These preparations were highlighted based on the operating room nurses’ experience of earthquake disaster preparedness in the face of the event.

##### Prediction of equipment resources

The participants discussed the challenge of forecasting and storing various medical and surgical equipment, required supplies, and personal protective equipment, which are important factors for operating room nurses’ earthquake disaster preparedness. The participants emphasized the importance of anticipating and preparing for disaster emergency situations by having a depot of equipment and surgical and medical supplies. The lack of available equipment resources was identified as a significant challenge that made rescue work difficult in the recent earthquake experience. Without adequate equipment resources, disaster preparedness efforts would be meaningless. Participants said:

*“In preparation for disasters, we predicted the equipment needed in advance, particularly in the operating room, and prepared some equipment for use in the event of disasters. Calibration and periodic visits were scheduled for repair or purchase of necessary equipment. Devices such as suctions and resuscitation trolleys were placed in a specific shelter to increase capacity in case of disasters. The operating room had already prepared a series of dedicated surgical sets”. (Participant N.3, woman with 28 years of work experience)*

*“In the operating room, several tasks had to be performed, including the supply and storage of all sterile equipment such as sterile surgical instrument sets, dressings, sterile gauzes, and suture sets for the hospital and the operating room itself”. (Participant N.4, woman with 25 years of work experience)*

##### Challenges of equipment and surgical instruments in disasters

Operating room nurses who participated in the study identified several equipment challenges in disasters, including the deficiency of available medical and surgical equipment, the management of surgical instruments according to the number of patients, long distances to access medical equipment, and the challenge of sterilizing surgical instruments. The experience of facing such equipment challenges limited the ability of operating room nurses to provide care. Some participants stated that working with instruments and surgical equipment is an important part of the duties of operating room nurses in different conditions, which becomes even more important in disaster situations. They described:

*“One of the challenges faced was the lack of equipment and facilities, which hindered the ability to effectively perform necessary tasks”. (Participant N.2, woman with 6 years of work experience)*

*“In the temporary hospital built after the earthquake, there were a few small ultrasound devices that no one knew how to operate. There were only 4–5 devices available. Additionally, there were a number of pedal-run suctions that required knowledge of their operation, as they did not run on electricity. The staff of other organizations were also unfamiliar with their use. These factors highlight the importance of considering the usefulness of equipment in critical conditions. Although they may not be useful at present, they can be highly practical during disasters. In such situations, the minimum number of instruments should be used in a maximally effective manner”. (Participant N.6, man with 29.5 years of work experience)*

*“In the surrounding area, several people were observed suturing with instruments, but it was unclear whether the instruments were sterile or not. There was no solution for sterilization available, and ordinary people did not know how to properly sterilize instruments. For example, even if a small part of the suture instrument touches something else, it should be sterilized before being used for further suturing”. (Participant N.13, man with 10 years of work experience)*

#### Managerial - organizational preparations for disasters

This category encompasses the operating room nurses’ descriptions of managerial and organizational preparations for disaster preparedness. The participants emphasized several concepts in disaster preparedness, such as the importance of operational planning, specific disaster guidelines, incident command systems, operational leadership, resource management, coordination, communication, and teamwork. The subcategories are described below.

##### Access to disaster operational plans and guidelines

The participants identified various challenges related to disaster preparedness due to deficiencies in management planning. Some participants suggested that the availability and continuous updating of dedicated operational disaster plans for the operating room unit and its staff can ensure disaster management is carried out in a secure framework. Based on the participants’ experiences, they proposed the necessity of designing and making available operational defined plans for different disasters, including earthquakes, specific guidelines for different phases of disasters to define the expected actions of the operating room unit along with other hospital departments, and the readiness of a predetermined manpower plan for disaster situations. However, some participants noted that despite the preparation of various guidelines and programs, they observed operational weaknesses and disruptions in the organization of earthquake relief. This was due to the fact that disaster conditions are such that the implementation of programs may not be carried out properly. Participants stated that:

*“Regrettably, the disaster plans that were made were only theoretical and not practical. The plans should be operational and practical, and when put into practice, they should be effective. Unfortunately, the plans were not long-term and were short-lived, with limited scope. Instead of being a team management approach and using the participation of all types of medical staff in long-term planning, the plans were often limited in scope”. (Participant N.14, man with 27 years of work experience)*

*“Different plans for disasters are developed at both national and local levels. These plans outline the necessary actions to be taken in each event, including who should perform each task and how. The plans thoroughly describe current conditions and make predictions for various scenarios, such as floods, earthquakes, fires, oxygen cutoffs, and infection diseases. Additionally, there is a plan called the increased capacity plan, which was implemented during the earthquake and detailed in a specific protocol. The first task in this plan is to reinforce emergency preparations”. (Participant N.15, woman with 14 years of work experience)*

##### Incident command system and in scene leadership

Operating room nurses emphasized that management and operational leadership of the complex are crucial in all phases of disaster preparedness, including disaster prevention, response, and recovery. As discussed in their experiences, leadership and structured management in decision-making and field management were identified as challenges in earthquake disaster preparedness.

The incident command system was identified as a crucial structure in the disaster preparedness of different hospital groups, including operating room nurses. The participants emphasized that the incident command system, with its standard and pre-planned management structure, plays an important role in managing and organizing necessary measures for disaster preparedness. However, in the Kermanshah earthquake in Iran, the performance of the incident command system was not effective in managing and organizing the situations. The interviews revealed that the lack of proper resource management in disaster situations has led to challenges in disaster management, including delays in relief delivery. The special role of the incident command system was discussed in almost all the interviews in terms of management, organization, and leadership of disasters and the preparation of healthcare teams for disasters. By clarifying the responsibilities of staff in this system, the unity of command for disaster management and control of situations can be facilitated. For example, participants stated that:

*“The management during the disaster was inadequate, with individuals acting in their own self-interest. There was a lack of field management, and orders were given from Tehran to go to places where there was no need to go. we were sent to places where we were unsure of what was waiting for us. In my perspective, it is crucial for the commander and leader to be present in the field rather than in a distant city giving orders”. (Participant N.9, man with 18 years of work experience)*

*“The disaster chart clearly outlines the chain of command and who is responsible first. For instance, the head of the hospital serves as the incident commander, followed by the deputies and management parts, with each management having sub-departments. This structure ensures that orders are given according to the needs of the situation, allowing for structured management and regular, controlled performance”. (Participant N.7, man with 23 years of work experience)*

##### Coordination in disaster management

The participants emphasized the importance of coordination and cooperation among healthcare staff and organizations in disaster preparedness. In their experiences during the earthquake, operating room nurses discussed the lack of coordination and cooperation among organizations, units, and individuals, as well as several sources issuing orders. Participants also discussed challenges in disaster management, including poor organization and preparation, slow response times for caring for earthquake victims, conflicts, parallel work in decision-making, and difficulty controlling irregularities. In this regard, the participants stated:

*“During the earthquake, confusion and parallel work were observed. For instance, when a certain medicine was needed, three cars were sent to retrieve it. Participants emphasized the need for a designated manager to coordinate and a coordination leader during pre-disaster planning and the disaster itself”. (Participant N.6, man with 29.5 years of work experience)*

*“During the earthquake, the presence of multiple decision-making centers caused confusion in the deployment of rescue efforts in the affected area”. (Participant N.9, man with 18 years of work experience)*

##### Effective communication and teamwork

According to some participants, it is important to prepare special teams for disasters, either in the form of an internal team within the operating room or as special multidisciplinary disaster preparedness teams. This is necessary for a coordinated and prompt response, as well as teamwork measures during the disaster response phase. The interviewees emphasized the importance of regular and direct communication at both the individual and organizational levels, including among personnel, in the mission rescue team, with upper managers, their own organization, and other organizations. Establishing communication systems for better management performance in a team group is also crucial for operating room nurses to effectively deal with disasters. Participants spoke:

*“Another experience gained from the earthquake is the importance of speed and communication. All sections, including different staff and the emergency unit, should work together and maintain communication without hesitation. Therefore, the role of communication is crucial. During a disaster, proper communication with staff from other organizations can facilitate effective activities. However, communication was not at a good level during the earthquake rescue”. (Participant N.12, man with 15 years of work experience)*

*“I was initially sent to the earthquake site but was later replaced by staff from Sanandaj in my workplace. operating room nurses, surgeons, and anesthesiologists from the same hospital should have stayed at the site because they are familiar with each other’s and each other’s abilities and skills, allowing for better communication and teamwork. We can work and communicate better with the team we already know. The expeditionary staff, who were not familiar with each other, made mistakes and had difficulty locating necessary equipment. As a resident and a member of the local team, I am familiar with the team’s abilities and skills. If the same team had been present, we would have faced no problems or deficiencies. However, taking the team’s human resources away from the hospital caused problems in managing the hospital’s situations during the disaster”. (Participant N.9, man with 18 years of work experience)*

#### Clinical skills for responding to disasters

This category encompasses the participants’ statements regarding the proper execution of various clinical skills, including triage skills, specialized operating room procedures, and basic nursing skills, in the context of earthquake disasters. The following subcategories were identified:

##### Triage skills

The participants emphasized the importance of proper triage skills in providing care services in response to earthquake disasters. Triage and prioritization of victims are considered crucial disaster response skills for nursing groups. Operating room nurses and other medical team members must have the skills to prioritize the treatment of different types of disaster victims with varying levels of injury based on the amount of damage sustained. This allows for the determination of necessary care based on the urgency of the victims’ needs. It was noted that if triage is not performed skillfully, it can cause disorder and further harm to the injured. Participants stated that:

*“Triage is the most important aspect to consider when responding to a disaster. It is necessary to prioritize the wounded based on the degree of injury sustained and the level of threat to life. Those in serious danger should be given priority”. (Participant N.2, woman with 6 years of work experience)*

*“We triaged a number of injured people on the hospital campus. We divided a number of nurses to attend to the injured people on the campus. A group of critically ill injured people received care in the emergency room. Tents were set up on the campus to treat some outpatients, and we also triaged patients who were in shock or had superficial injuries. This approach made it much easier to care for the injured”. (Participant N.14, man with 27 years of work experience)*

##### Special skills as an operating room nurse

Operating room nursing requires a set of specialized skills related to occupational expertise to implement life-saving measures for groups of victims in disasters. According to the participants, these skills include ligation and stopping bleeding, suturing, assisting in various emergency surgeries, helping to stabilize limb fractures and splinting, and observing and maintaining sterilization of the surgical field. The participants discussed these skills in the context of their experiences during the earthquake. Many participants highlighted the importance of previous experience in emergency situations and rescue work in disasters as an effective factor in the disaster preparedness of operating room nurses. This is because operating room nurses perform necessary skills in these situations similar to those required during disasters. In this regard, the participants stated:

*“In my opinion, operating room nurses play an important role in providing assistance during disasters, as observed during the earthquake. For example, they can assist with suturing, splinting, administering intravenous injections, cleaning injury sites, and providing emergency surgical sets. Emergency procedures in the operating room, such as ruptured spleen, intra-abdominal bleeding and hematoma, and shock, are also within the purview of operating room nurses”. (Participant N.1, man with 11 years of work experience)*

*“Operating room nurses play a crucial role during disasters and must be able to provide care at a high level. During a disaster, operating room nurses should prioritize maintaining sterility and performing specialized duties with speed”. (Participant N.4, woman with 25 years of work experience)*

*“We had 20 beds arranged side by side on the hospital campus and were able to hospitalize 20 wounded individuals. We treated each patient in turn, performing basic procedures such as suturing for those with bleeding and cuts, and preparing splints and fixators for those who could be transferred to provincial hospitals. My experience working in the trauma center of the province was helpful, as we often cared for 200–300 traumatic patients daily with minimal resources and facilities”. (Participant N.13, man with 10 years of work experience)*

##### Basic nursing care skills

The participants discussed the importance of basic nursing skills required to perform routine treatment procedures, which nursing groups, including operating room nurses, must possess. They noted that in disaster situations, they may be required to provide assistance as nurses in the nursing department, which can be a challenging task. Based on their experiences, the participants mentioned performing nursing procedures such as first aid, cardiopulmonary resuscitation (CPR), dressing, bandaging, and initial wound control as basic nursing skills necessary for earthquake disaster measures. For instance, participants stated:

*“When we arrived, the disaster victims had not yet been brought to the operating room, and the emergency room did not have enough nurses. We went there to assist with outpatient procedures that could be done in the emergency room. It was noted that everyone should have the ability to perform resuscitation and know CPR, as these are basic skills”. (Participant N.8, man with 17 years of work experience)*

*“Operating room nurses have several duties to perform during times of disaster. If we only act as operating room nurses, our helping role will be limited. Therefore, we should also be able to perform other nursing skills”. (Participant N.7, man with 23 years of work experience)*

#### Resilient ability in disaster response situations

Resilience in challenging disaster response situations emerged as a main category in many interviews. This category included three subcategories: ethical conflicts in care provision, adaptation to difficult environmental and occupational conditions during disasters, and maintaining the calmness and mental well-being of operating room nurses during disaster response situations.

##### Ethical conflicts in care provision

Several participants identified personal and professional ethical challenges faced by operating room nurses during earthquake disaster care provision. Conflicts arose between adhering to operating room standards and principles and ignoring some or all of them during the disaster response phase. Compliance with infection control principles, equipment and supply deficiencies for treating the injured, and ethical decision-making regarding their use for disaster victims were also discussed as ethical conflicts in disaster response conditions. These ethical challenges can impact the treatment and survival of the wounded, as well as the personal conscience, responsibility, and safety of operating room nurses. Therefore, solutions and strategies should be considered to overcome such situations. Participants emphasized the need for:

*“One of the problems we faced was that, according to the principles, we were not allowed to go to other departments while wearing operating room clothes. However, due to the lack of staff and the emergency situation, we had to go to the emergency room wearing the same clothes. When we returned, our clothes were no longer sterile. Despite this, we had to prioritize life and death over sterility”. (Participant N.5, woman with 10 years of work experience)*

*“During the earthquake disaster, we had 20–30 general surgical instrument sets. However, due to the high number of patients, we had to use two instruments from each set for one patient. This meant that we used two instruments for each patient, instead of having 20 sets for 20 patients. In normal conditions, care should be taken to maintain sterility, and one set should be used for each patient. However, in disaster conditions, we had to divide one set among 10 patients”. (Participant N.6, man with 29.5 years of work experience)*

##### Adaptation to difficult environmental-occupational conditions in Disasters

The majority of participants in our study emphasized the importance of being prepared to adapt to difficult conditions and take initiative when responding to an earthquake disaster. Participants described the challenging situations they faced during the earthquake response, including harsh environmental conditions, limited food resources, inadequate living facilities, lack of proper shelter, heavy workload, and sleep deprivation. They stressed the need to facilitate such conditions during disasters. Based on their experiences, continuous work shifts and long hours can lead to energy depletion, job burnout, and medical errors among operating room nurses. Therefore, appropriate strategies such as inter-personnel rotation and sufficient human resources should be implemented to maintain the ability to provide care in disaster conditions. Additionally, individual adaptation abilities and creativity in performing non-therapeutic measures should be considered. One participant stated:

*“In my experience during the earthquake, I believe that those who are present to help should have an adaptable personality. I witnessed people from other cities with different languages and cultures who were unable to provide effective assistance. Skilled staff must be able to adapt to difficult conditions. In disaster situations, basic or ideal facilities such as drinking water, sanitary facilities, physical hospital units, and tents may not be available. Everyone should not wait for facilities but instead work in other areas and even outside their area of expertise to provide assistance. For example, we helped set up tents, supply water, provide plumbing, and cook food. Therefore, in the event of a disaster, an operating room nurse must be creative and helpful”. (Participant N.1, man with 11 years of work experience)*

##### Maintaining mental well-being in disaster situations

The participants in the study perceived it as critical to remain calm during earthquake disaster response in order to properly perform their duties. Following the earthquake, people in the affected area were extremely stressed, which posed a challenge for the participants. They also expressed concerns for their family members in stressful conditions, exposure to difficult situations, and increased work pressure due to the influx of disaster victims. Despite these challenges, the participants emphasized the importance of remaining calm and providing first aid to the victims as an operating room nurse. They also suggested that training and preparing family members during the prevention phase could reduce stress for operating room nurses during disaster rescue work, thereby increasing their disaster preparedness. The participants noted:

*“After the earthquake, the hospital corridors and operating rooms were crowded with patients. In such tense and crowded conditions, if counting is not done carefully, an instrument may be left behind, or complications may arise during another surgery for the patient. Therefore, it was crucial to remain as calm as possible in these situations. If one’s mind is disturbed or stress affects the person, these challenges can make it difficult to control the situation. This can have adverse effects on the patients and cause everything to get out of control”. (Participant N.5, woman with 10 years of work experience)*

*“It is important for operating room nurses to remain calm, as some may become easily upset when faced with child casualties or children who have lost their entire families. Many staff members brought their families with them because they had nowhere else to go, and they were constantly worried about them. The families resided in their cars in the parking lot while the staff worked”. (Participant N.11, man with 30 years of work experience)*

## Discussion

The current study investigated the disaster preparedness experiences of operating room nurses involved in healthcare provision after the 2017 earthquake in Kermanshah, Iran. A qualitative approach was employed, using semi-structured and in-depth interviews to identify and explain the topic of earthquake disaster preparedness. Through analysis of the results of this qualitative study, six categories were identified: knowledge and perception of preparedness for disasters, educational and training programs for disaster preparedness, equipment preparedness for disasters, managerial and organizational preparedness for disasters, clinical skills for responding to disasters, and resilient ability in disaster response situations.

The findings indicate that the knowledge level of operating room nurses is a crucial factor in their disaster preparedness. Moreover, it is underscored that nurses must possess knowledge competence to deliver appropriate care during disasters and critical conditions [46–48]. The inadequacy of knowledge and skills in disaster management implies that nurses are insufficiently prepared [32]. As previously noted, operating room nurses who lack adequate knowledge of disaster management may encounter difficulties in performing their expected duties and skills during actual earthquake disasters. The importance of knowledge-based nursing measures in disaster management is also highlighted [49]. The ambiguity surrounding the roles of nurses posed a significant challenge in disaster management [50]. Another study demonstrated that knowledge sharing should align with the roles and responsibilities of nurses in disaster management, as well as the hospital protocols [51]. Enhancing the knowledge level of operating room nurses, their perception of disaster program components, and personnel description duties in disasters can lead to an improvement in their disaster preparedness. Simulations can enhance nurses’ knowledge and increase their awareness of their responsibilities during the disaster response period [32, 52]. Offering standardized and ongoing education can be an effective approach to enhancing the knowledge of operating room nurses in earthquake disaster management and preparedness. Such education can improve participants’ disaster knowledge and awareness of their roles and duties during various phases of disasters.

In interviews, operating room nurses identified the lack of comprehensive educational preparation for earthquake disaster preparedness as a significant challenge. The absence of adequate training has created numerous obstacles for nurses and healthcare workers during recent epidemics [53]. Adequate training for nurses can have a positive impact on the outcomes of disaster victims [54]. In Iran and several other countries, disaster preparedness programs are inadequately integrated into university nursing curricula [2, 55]. The development and implementation of disaster educational exercises and drills in hospitals can be beneficial in preparing operating room nurses for such emergencies. Consistent with our findings, studies have emphasized the significance of education and training in enhancing nursing preparedness for disasters [56–59]. Improper execution of such exercises may pose challenges for hospitals during disasters. Participants have identified challenges such as the lack of seriousness in providing training programs and exercises, their inadequacy in continuity and applicability to actual disaster conditions. The absence of evaluation and feedback of the education was also reported as a challenge in the Goniewicz and Goniewicz study [60]. Policymakers and hospital managers can enhance the preparedness of operating room nurses for earthquake disasters by providing them with specific, standardized, and practical training through continuous interdisciplinary education, from university to in-service training, using innovative formats. Sufficient disaster training can enable operating room nurses to make effective contributions during various phases of disaster preparedness.

In the aftermath of the Kermanshah earthquake in Iran, many participants emphasized the significance of having access to medical equipment to carry out their duties as operating room nurses. Operating room nurses may encounter challenges in obtaining essential equipment and supplies, including disposable equipment, surgical sets, and sterile surgical instruments. The preparation of surgical instruments is a crucial aspect of the job requirements for surgical nurses in disaster response [61]. Likewise, other studies have highlighted the importance of equipment and resource preparedness for disasters [30, 61]. Adequate equipment preparation is crucial not only in storage and support areas but also in the ability to use the equipment to achieve optimal preparedness during prevention and response phases. Following disasters such as earthquakes, surgical equipment must be promptly prepared [62]. The importance of having access to appropriate personal protective equipment to ensure the safety of both nurses and disaster victims from transmissible diseases was emphasized. This finding is consistent with another study [60]. Nevertheless, our study identified challenges in working with surgical equipment during the response to the Kermanshah earthquake, such as the limited number of instruments and the sterilization of instruments. The issue of limited access to instruments during disasters was also reported by Abdi et al. [33] Additionally, inadequate equipment during disaster situations can disrupt the process of rescuing patients [63]. The Insufficienty of equipment during disaster situations significantly limits nurses’ ability to provide nursing care [32]. Another study emphasized the challenge of performing nursing procedures with minimal available materials and personnel [49]. To address such challenges, reinforcing individual management in equipment usage and storage of appropriate equipment reserves for similar disasters can be beneficial. Preparedness for equipment usage, resource storage, and ensuring these processes can be considered as strategies for earthquake disaster preparedness of operating room units and even entire hospitals. This can improve hospital readiness for any type of emergency situation and possible disasters.

The present findings revealed another main category extracted from the interviews, which was the experiences of operating room nurses in managerial and organizational preparations. Operating room nurses emphasized the significant role of the disaster management system in disaster preparedness command, and its defective structure can lead to problems such as poor leadership of the disaster scene and inappropriate management of resources. The inadequate management of resources has had an impact on the survival of those affected by the Kermanshah earthquake [64]. In the context of Hurricane Sandy rescue experiences, nurses identified leadership as a key factor in adapting to challenges [50]. Li et al.‘s study identified coordination and leadership as the primary factors related to nurses’ experiences during the Wenchuan earthquake [29]. Consistent with our findings, another study emphasized the significance of command and control in disaster management [65]. Hospital managers and disaster commanders can design strategies for future disaster management by prioritizing inter-organizational coordination and communication in disaster preparedness, including timely presence, coordinated access to resources, organized relief, and effective communication and teamwork. The importance of communication has been highlighted as one of the lessons learned in the recent pandemic [66]. Inadequate coordination and communication between organizations can disrupt the process of disaster preparation for nurses and hinder their response to disasters.

Operating room nurses emphasized the importance of disaster protocols and planning, as well as the existence of an integrated incident command system, in disaster preparedness. Proper coordination through the disaster management system can have a positive impact on disaster preparedness and management [30]. Disaster protocols can aid in managing processes during disaster preparedness [67–69]. The availability of pre-prepared plans and guidelines that specify coordination, communication, and detailed positions of medical staff for disaster situations, as well as having strong and always-ready hospital disaster management systems, can enhance the readiness of health staff for disaster preparedness. However, our study identified the challenge of not implementing plans for the disaster scene. In this regard, the significance of developing organizational strategies and operational guidelines to support nurses during the stages of disaster preparedness has been emphasized [48, 70]. The absence of protocols and preparedness programs resulted in significant damages and injuries during the Wenchuan earthquake [29, 71]. Comprehensive structuring and organization in management areas, as well as addressing management challenges, can be incorporated into the process of disaster planning and management to enhance the disaster preparedness of the health services system.

According to the participants, possessing an appropriate level of practical and clinical skills for providing clinical care is a crucial component in providing an appropriate response during disasters. Previous work experience in disasters was identified as an important factor for disaster preparedness. It has been demonstrated that work experience and history of care provision during disasters may play significant roles in nurses’ skills [72, 73]. Participants emphasized the importance of performing clinical skills with speed, accuracy, and in high volume, along with more specific skills for disaster situations. In this regard, participants highlighted specialized skills required of operating room nurses, such as suturing, observing principles of infection control, and maintaining sterility, as well as triage skills and basic nursing care skills. It has been noted that nurses perform these skills daily in challenging situations [35]. This finding is consistent with other studies that have identified a range of clinical skills required for disasters, including triage and primary care, as well as more advanced skills for the care of trauma patients [74, 75]. Strengthening the aspect of skills competencies has been shown to further develop nurses’ disaster preparedness [47]. Previous studies have also highlighted basic life support skills and triage as essential skills for responding to disasters [76]. Developing educational programs to enhance the skills of nurses in the field of disaster preparedness is essential [77]. Despite the significance of providing psychological support for victims of disasters [75], Operating room nurses perceived providing psychological support during disasters as outside the scope of their skills and identified it as a role for other specialists. Promoting and emphasizing clinical skills as one of the most important skills, particularly for novice nurses, through various methods can ensure the disaster preparedness of operating room nurses and contribute to saving the lives of victims of earthquake disasters.

Another finding of the present study was the resilient ability of nurses in earthquake disaster response situations. Participants highlighted unfavorable conditions, such as problems with nutrition, ethical conflicts, long and continuous working hours, difficult environmental conditions, physical exhaustion, and stress, which can distract their attention from providing an adequate response to disasters. Other studies have shown that nurses may face difficult environmental conditions and a lack of facilities during disasters [78]. Prolonged high workload pressure during disaster situations can lead to fatigue and burnout, which can decrease nurses’ productivity [79]. Adapting to these conditions and demonstrating resilience during difficult times can contribute to better disaster preparedness among operating room nurses. Following challenging disaster situations, individuals are often confronted with the need for ethical decision-making, and in some cases, such as during the recent earthquake, they may have to disregard professional or social principles. Therefore, it is crucial for nurses to be adequately prepared to manage safety risks and moral dilemmas when dealing with disasters [80, 81]. Greco et al. [82] has emphasized the need for educational support for nurses to prepare them for ethical decision-making. The study findings revealed that operating room nurses experienced difficult mental conditions, concerns for their families, and various forms of stress in response to the Kermanshah earthquake disaster in Iran. The psychological well-being of nurses can be affected by their presence in disaster situations due to factors such as job pressure and personal concerns [31, 75, 83, 84]. Li et al. found that nurses highlighted their lack of physical and mental preparation when being sent to the disaster-affected area of the Sichuan Ya’an earthquake [29]. Preparing operating room nurses to develop the skills needed for resilience in such situations, along with a background in disaster preparedness, can be considered to enable them to effectively provide care for victims of disasters during such events. Yuan et al. [85] identified resilience strategies in the resilience trajectories of front-line nurses during disasters. Mental and physical preparation, as well as personal planning for potential disasters, may enhance performance in such situations.

### Strengths and limitations of the study

One limitation of the present study is that it was conducted in only one province (Kermanshah) during one disaster (the 2017 earthquake in Kermanshah, Iran). Therefore, due to the nature of qualitative studies, our findings may not be generalizable to hospitals in other provinces or for all disasters. Despite these limitations, this study provides clear evidence about the disaster preparedness of operating room nurses, which can be used in future planning and policymaking to enhance the disaster preparedness of operating room units and improve outcomes for disaster victims. During some interviews, other people entered the interview location and disrupted the participants, in which cases the researcher politely asked them to leave the participants’ interview space. In some cases, participants did not speak clearly due to concerns about the possibility of conversations being disseminated and the negative effects of their opinions on their job conditions. The researchers tried to ensure participants that the information would not be disclosed and conducted interviews during favorable and quieter hours and working shifts. There may also be concerns about the limitations of recall bias due to the interval time between the event and the interviews. However, the rich and strong codes and categories extracted from the interviews can overcome this limitation.

## Conclusion

The present study utilized a qualitative approach to explore the experiences of operating room nurses who were involved in rescue efforts during the 2017 earthquake in Kermanshah, Iran, with regards to earthquake disaster preparedness. One main theme and six main categories were extracted from the experiences of Iranian operating room nurses. The findings of this study can be used by hospital and nursing managers, policymakers, and operating room nurses to take necessary measures to prepare for earthquake disasters. The experiences highlighted in this research and other similar studies can be used to solve challenges and facilitate barriers by providing systematic strategies, leading to better preparation and fewer challenges for operating room nurses during such disasters. Hospital managers can apply operational strategies to manage all phases of disasters by providing in-service training and exercises, considering the facilitation of human resources, support, and equipment. Actions can also be taken to improve the clinical skills and individual resilience of operating room nurses during disaster response, thereby improving their level of earthquake disaster preparedness. It is necessary to carry out experience-based plans and strategies to reduce the adverse effects of deficiencies in disaster preparedness, such as injuries to patients, damage to operating room nurses’ capabilities, damage to the medical system, and society in possible future disaster conditions. It is suggested that studies be conducted to examine strategies and interventions aimed at improving the level of disaster preparedness among operating room nurses. Future studies should aim to provide a deep understanding of disaster preparedness among other healthcare staff.

### Supplementary Information

**Additional file 1.**

## Data Availability

The datasets used and analysed during the current study are available from the corresponding author on reasonable request.
