# Supplementary material for: Experiences of operating room nurses in disaster preparedness of a great disaster in Iran: a qualitative study
Source: BMC Emerg Med. 2023 Nov 23;23:138. doi: 10.1186/s12873-023-00903-w (PMC10668482; doi:10.1186/s12873-023-00903-w)
Supplement: Supplementary file 1 — Additional file 1. [file 12873_2023_903_MOESM1_ESM.docx]

**Interview Guide**

**Type of questions**

Questions

**Introduction**

Introduction of the main researcher

Explaining the objectives and importance of the study

Voluntary participation in the study

Use of data anonymously

No right or wrong answer and explain your opinion in every question

Obtaining verbal and written informed consent

**Beginning**

Demographic information questions

Please describe your experience of participating in the disaster preparedness activities In the Kermanshah earthquake?

Please describe your experiences during relief operation of Kermanshah earthquake?

What have you learned from experiences during disaster preparedness efforts?

What challenges did you experience before, during and after your work as an operating room nurse in the disaster preparedness activities In the Kermanshah earthquake?

What do you do to deal with problems that encounter?

Based on your experiences, what components and features should be considered in the earthquake disaster preparedness of operating room nurses?

Please tell me about the expectations and obstacles of current status of earthquake disaster preparedness?

Further questions based on the objectives of the study and answers of the participants.

**Development and probing**

Including probing questions

Can you please give an example of this?

Could you please explain with more details?

What do you mean by that?

**Closing**

Are there any untold things about the disaster preparedness of operating room nurses In the Kermanshah earthquake?

Is there anything else you would like to talk about it?

Supplementary legends:

After identifying the potential participants who met the inclusion criteria, they were invited to participate in the study, and after explaining the study objectives and obtaining the initial agreement, verbal and written informed consent was obtained from participants, and subsequently an interview appointment was set. To collect data, semi-structured and face-to-face interviews were held with considering data saturation. The first author, M.R., interviewed all participants individually, under the supervision of supervisors in the research team who were experts in qualitative research methods. The interviews were conducted confidentially, in a private and quiet space, usually in the rest room of the operating room staff, and at a suitable appointment at the desired time for the participants. Written field notes were taken by the Interviewer during the interviews to be used for a correct description and interpretation of the data. The interview guide used in present study was developed for this study and include Introduction, Beginning, Development and probing, Closing sections. The interview guide questions were designed by the research team based on the objectives of the study, according to the literature review and with considering qualitative descriptive methods. Also before the main interviews, two pilot interviews were conducted to ensure the adequacy of the interview questions for achieving the study's objectives. The pilot interviews showed that the interview questions were appropriate to achieve the objectives of the study. The general interview questions were designed after minor revisions. At the beginning of the interview, demographic questions (such as gender, age, educational level, marital status, and years of work experiences) were asked, and then the interview schedule started.
